# Economic evaluation of tenofovir disoproxil fumarate prophylaxis to prevent mother-to-child transmission of Hepatitis B virus infection: evidence from a lower-middle income country

**DOI:** 10.1186/s12913-024-12152-z

**Published:** 2024-12-28

**Authors:** Ha T. Nguyen, Usa Chaikledkaew, Minh V. Hoang, Viet Q. Tran, Montarat Thavorncharoensap, Naiyana Praditsitthikorn, Quang D. Tran, Ammarin Thakkinstian

**Affiliations:** 1 Mahidol University Health Technology Assessment (MUHTA) Graduate Program, Mahidol University, Bangkok, 10400 Thailand; 2 University of Health Sciences, Vietnam National University Ho Chi Minh City, Binh Duong City, 820000 Vietnam; 3 Social and Administrative Pharmacy Division, Department of Pharmacy, Faculty of Pharmacy, Mahidol University, Bangkok, 10400 Thailand; 4 Hanoi University of Public Health, Hanoi, 100000 Vietnam; 5 Military Hospital 175, Ho Chi Minh City, Vietnam; 6 Division of Innovation and Research, Department of Disease Control, Ministry of Public Health, Nonthaburi, 11000 Thailand; 7 Communicable Disease Control Division, General Department of Preventive Medicine, Vietnam Ministry of Health, Hanoi, 100000 Vietnam; 8 Department of Clinical Epidemiology and Biostatistics, Faculty of Medicine Ramathibodi Hospital, Mahidol University, Bangkok, 10400 Thailand

**Keywords:** Economic evaluation, Mother-to-child transmission, Hepatitis B, Preventive strategies, Vietnam

## Abstract

No cost-effectiveness information of preventive strategies for mother-to-child transmission (MTCT) of hepatitis B virus (HBV) has existed for policy decision making. This study aimed to compare the cost-effectiveness of alternative strategies to prevent MTCT of HBV in Vietnam. Cost-utility analysis using a hybrid decision-tree and Markov model were performed from healthcare system and societal perspectives. Preventive strategies included HBV universal vaccination (S1), hepatitis B immunoglobulin (HBIG) for infants of mothers with HBeAg( +) (S2), HBIG for infants of mothers with HBsAg( +) (S3), tenofovir disoproxil fumarate (TDF) for mothers with high viral load (S4) and mothers with HBeAg( +) (S5), and the current practice (S6) with HBV vaccine in all infants and TDF for high viral load mothers. The current practice was dominant to all preventive strategies with interventions in only infants for both perspectives. In contrast, the strategies S4 and S5 were dominant to the current practice with incremental net monetary benefit varying from $33.94 to $70.64 under a healthcare system perspective and from $44.22 to $93.71 under a societal perspective. Addition of HBIG in infants born to mothers infected HBV and tenofovir prophylaxis for mothers with positive HBeAg was the most cost-effective strategy to prevent vertical transmission of HBV in Vietnam.

**Supplementary Information:**

The online version contains supplementary material available at 10.1186/s12913-024-12152-z.

## Introduction

Hepatitis B virus (HBV) is a major public health problem, with approximately 296 million with chronic hepatitis B (CHB) and 820,000 deaths in 2019 worldwide [1]. In high prevalence regions, mother-to-child transmission (MTCT) accounted for the major path of HBV transmission [2, 3], in which rate of CHB is noticeably very high among infants infected at birth or during the first year of life as compared to older population [4]. In 2016, elimination of viral hepatitis was set as the global public health threat, in which one important goal was to reduce the prevalence of hepatitis B surface antigen (HBsAg) among children under 5 years to 0.1% [5].

The efficacy of HBV vaccine, hepatitis B immunoglobulin (HBIG) in infants, and antiviral-agents of tenofovir disoproxil fumarate (TDF) for MTCT prevention has been proven in previous meta-analyses and network meta-analyses of randomized clinical trails (RCTs) [6–9], and these have been recommended in clinical practice guidelines [10–13]. Although the TDF prophylaxis is recommended in mother with high viral load (VL) in most guidelines [10–12], this may not be accessible in resource constrained settings due to its high cost and high-technology requirements of HBV DNA quantitative test. To overcome this barrier, since 2020 the World Health Organization (WHO) [13] has recommended the usage of HBeAg test as a proxy for high VL to improve accessibility and reduce inequities across population.

Vietnam was one of countries with high prevalence of HBV, approximately 10.5% in adult population and 10.8% among pregnant women [14]. Since 2018, the Vietnam Ministry of Health has issued the action plan to eliminate MTCT of HBV by 2030. The combination of HBIG and HBV vaccine birth dose (BD) in infants of HBsAg( +) mothers has been recommended [15], and TDF prophylaxis should be provided for pregnant women with high VL. Currently, HBV vaccine has been provided in the National Expand Program on Immunization (NEPI), and TDF has been included in the health-coverage scheme. However, HBIG has not yet been covered and patients must bear the cost of $72 per dose by out-of-pocket [16]. Consequently, only less than 7% of patients could get access to HBIG [17]. Furthermore, HBV DNA quantitative test is available and reimbursed only in provincial or higher level of hospitals, while HBeAg test can be reimbursed in lower level of hospitals. Therefore, the usage of HBeAg test as a proxy of high VL, recommended by the WHO [13], is feasible to scale up prevention.

Evidence on cost-effectiveness is important to support the making of decision on which HBV MTCT preventive strategy should be implemented in Vietnam. Nevertheless, such evidence has not yet been found. According to our systematic review [18], most cost-effectiveness of HBV MTCT preventive strategies were performed in high-income countries (HIC) and only one study in the border of an upper-middle-income country (UMIC) and lower-middle-income country (LMIC), where medical services were provided in refugee camps. Therefore, we aimed to compare the cost-effectiveness of six HBV MTCT preventive strategies in order to provide evidence to support for policy decision making in Vietnam and other similar settings.

## Methods

### Target population

All pregnant women who were screened for HBsAg as per the WHO guideline [19]. This cost-utility analysis model allowed us to construct the whole birth cohort of newborns in 2022 and their mothers in Vietnam.

### Intervention and comparators

According to current guidelines [10–12] and WHO’s recommendations updated in March 2024 [20], six preventive strategies (S1-S6) were designed to provide preventive strategies in infants (i.e., HBV vaccination and HBIG) and in mothers (i.e., TDF administration). Table 1 presents details of each preventive strategy.

**Table 1:** Description of preventive strategies to prevent HBV mother-to-child transmission

Strategy number	Strategies name	HBV biomarker test	Intervention in infants		TDF prophylaxis in mothers
			**Vaccine**	**HBIG**	
S1	Universal vaccination	(-)	( +)	(-)	(-)
S2	**HBIG for infants of mothers with HBeAg( +)**	( +) HBsAg test HBeAg test	( +)	( +)	(-)
S3	**HBIG for infants of mothers with HBsAg( +)**	( +) HBsAg test	( +)	( +)	(-)
S4	TDF for mothers with high viral load	( +) HBsAg test HBV DNA	( +)	( +)	( +)
S5	TDF for mothers with HBeAg( +)	( +) HBsAg test HBeAg test	( +)	( +)	( +)
S6	Current practice	( +) HBsAg test HBV DNA	( +)	(-)	( +)

## Model structure

A hybrid decision tree and Markov model were constructed to compare costs and health outcomes of six preventive strategies considering both societal and healthcare perspectives. Average total costs and health outcomes were estimated during patients’ lifetime period with a cycle length of one year using a half-cycle correction. Local clinical experts were consulted to validate the model structure to assure that disease progression pathway was appropriate and also reflected local context.

**Fig. 1:**
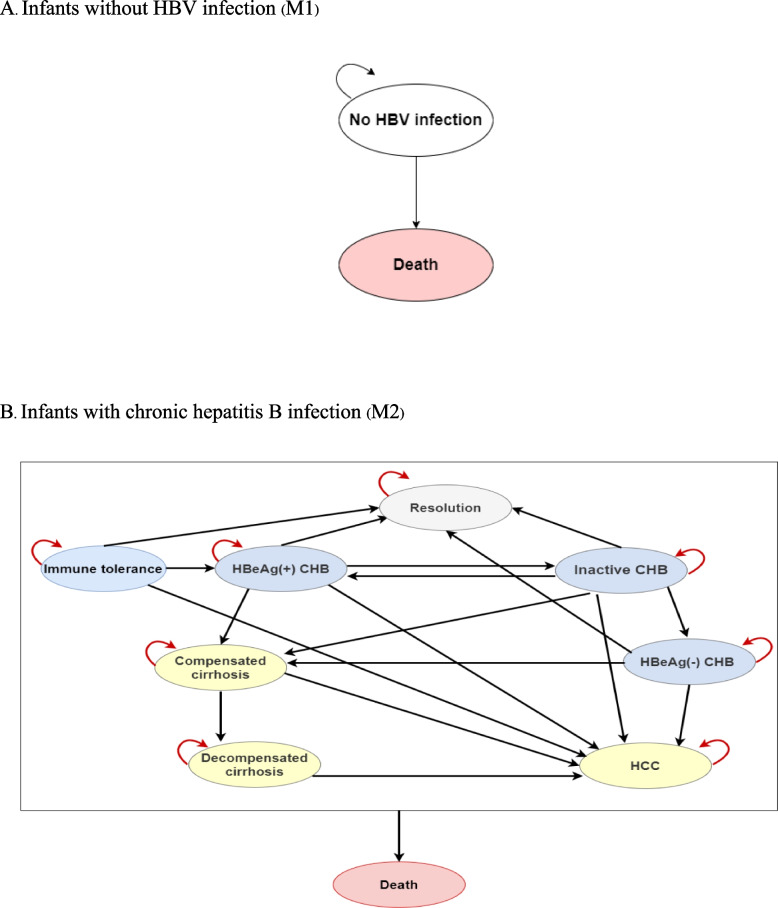
Markov model structure. HBV: Hepatitis B virus; CHB: Chronic hepatitis B; HCC: Hepatocellular carcinoma

All pregnant women entered the decision tree model under six preventive strategies (Figure S1). At the age of one year, the infants without HBV infection entered Markov M1, which consisted of two health states, i.e., living without CHB and death, see Fig. 1. Infants with positive HBV entered Markov M2, which illustrated lifetime progression of CHB patients due to vertical transmission consisting four non-cirrhotic CHB states and three CHB consequence states (i.e., compensated cirrhosis (CC), decompensated cirrhosis (DCC) and hepatocellular carcinoma (HCC)). In addition, the Markov M2 also included two other states, including resolution (HBsAg seroclearance) and death. Model was built and run using the Microsoft Office Excel 2016.

### Model assumptions

Infants vaccinated with HBV vaccine BD were assumed to complete three doses as per the NEPI, and vaccination would result in lifelong protection. This model ignored horizontal transmission, therefore newborns of HBsAg(-) mothers were assumed not to be infected. In addition, patients in the CC, DCC and HCC states were assumed to die due to liver related causes, while others had the same mortality rate as general population.

### Model parameters

#### Screening effectiveness and efficacy of interventions

The performance of HBsAg rapid diagnostic test (RDT) was obtained from a systematic review and meta-analysis which was performed to support for the WHO guideline on HBV testing [21]. Meanwhile, coverage of other HBV biomarker tests was obtained from the Department of Maternal Health and Children in Vietnam and expert consultations.

Preventive efficacy of different interventions measured by the percentage of HBV MTCT at age of 6–12 months were systematically synthesized by the team [6]. Due to the lack of data from RCTs, the efficacy of HBV vaccine in infants, HBV vaccine plus HBIG in infants of mothers with high and low VLs were assumed to be similar to those in infants of HBeAg( +) and HBeAg(-) mothers, respectively as per the WHO’s recommendations on the usage of HBeAg as a proxy of high VL.

#### Transition probabilities

Transition probabilities among different states in the Markov model were obtained from published literatures and consultation with local experts. The rate of all-cause mortality was obtained from the life-table of Vietnamese population via the WHO website [22]. Epidemiological parameters were retrieved from previous surveys in Vietnam [23].

#### Costs

Costs of HBIG and TDF were estimated from the bidding results of whole country in Vietnam in 2021. Meanwhile, costs of HBV biomarker screening tests were obtained from the National List of Medical Service in Vietnam in 2021. Direct medical cost (DMC) of non-cirrhotic CHB was estimated based on a retrospective primary data collection in a tertiary military hospital in Vietnam in 2020. Cost-at-charge approach was applied to convert all charges to cost values. Other cost components were borrowed from previous economic evaluations (EEs) [24, 25] in Vietnam. All costs were converted to the USD 2022 using Vietnam consumer price index [26] and exchange rate of $1.00 = 23,132 Vietnamese Dong.

#### Health outcomes

The 5-level EQ-5D (EQ-5D-5L) utility values of health states were borrowed from an observational study on 319 non-cirrhotic CHB patients [27] in China and the study [24] which collected primary data on CC, DCC and HCC patients in Vietnam. Both costs and outcomes were discounted at a rate of 3% based on Ministry of Health’s recommendation on EE reporting in Vietnam. Details on input parameters’ values were presented in the Table S1.

## Result presentation

The incremental net monetary benefits (INMB) of alternative strategies S1-S5 compared to the current practice S6 were estimated given the willingness-to-pay (WTP) threshold of Vietnam as follows: INMB = Incremental QALYs x WTP – Incremental costs. In this study, we used the WTP of one GDP per capita ($3,855) because the interested intervention strategies were for preventions, in which the WTP was usually lower than that of treatment interventions [28].

### Uncertainty analysis

One-way sensitivity analysis (OWSA) was performed to investigate the most influential parameters on the INMB. Dispersion of key parameters were estimated based on 95% confidence interval (CI). If the 95% CI was not available, a standard error (SE) of 20% of base-case value was applied. In addition, probabilistic sensitivity analysis (PSA) with 10,000 Monte Carlo simulations was also performed. Furthermore, due to the lack of robust data on the coverage of screening tests and prophylactic interventions, three different scenarios corresponding to targeted coverages varied from 50 to 95% for three consecutive durations from 2018 to 2030 according to the action plan issued by Vietnam Ministry of Health were analyzed [29].

## Results

### Costs and health outcomes

From healthcare system perspective, average costs of three strategies in infants only (i.e., S1, S2 and S3) varied from $47.40 to $48.85 per infant, which were higher than those of preventive strategies in both mothers and infants (S4-S6) accounting from $36.02 to $40.92, see Figure S2A. In addition, the two strategies with the addition of HBIG in infants of HBsAg( +) mothers and TDF prophylaxis in pregnant women (i.e., S4 and S5) incurred lower cost than the current practice S6 (accounted $40.92), which provided TDF prophylaxis in only mothers with high VL without HBIG in infants. Noticeably, CHB treatment cost accounted for the major proportion of total costs in all preventive strategies (Figure S2A). Similar trend was observed from the societal perspective. However, the addition of direct non-medical cost and time costs increased the average cost more than three times relative to healthcare system perspective costs (Figure S2B). Regarding the rate of CHB among 1-year-old children, the current practice resulted in a rate of 0.97%, as compared to 0.86% and 0.76% in strategy S4 (HBIG in infant of HBsAg( +) mothers plus TDF in only high VL mothers) and S5 (HBIG in infant of HBsAg( +) mothers plus TDF in only HBeAg( +) mothers), respectively. In contrast, all three S1, S2, and S3 in only infants led to higher rates of CHB, varying from 1.09% to 1.27%. The similar trend was observed in all scenarios with different coverages of prophylactic interventions (Figure S3).

### Cost-effectiveness analysis

For the healthcare perspective, all three S1, S2, S3 in only infants were dominated whereas the S4 and S5 were contrastingly dominant comparing with the current practice of S6. At the WTP of one GDP per capita, the INMB of S4 and S5 were $33.94 and $70.64, respectively, while the INMBs of S1, S2 and S3 varied from $−100.81 to $ −43.01. Similar trend was also found for the societal perspective (Table 2).

**Table 2:** Estimation of ICER and INMB comparing different strategies with the current practice

Prophylactic strategy	LY	Inc. LY	QALY	Inc. QALY	Cost ($)	Inc. Cost ($)	ICER	INMB ($)
**From healthcare system perspective**								
S1: Universal Vaccination	29.0606	−0.0097	28.9997	−0.0241	48.85	7.93	Dominated	−100.81
S2: HBIG for infants of mothers with HBeAg( +)	29.0644	−0.0059	29.0092	−0.0146	47.40	6.48	Dominated	−62.67
S3: HBIG for infants of mothers with HBsAg( +)	29.0665	−0.0038	29.0143	−0.0095	47.46	6.54	Dominated	−43.01
S4: TDF for mothers with high viral load	29.0738	0.0035	29.0326	0.0088	40.83	−0.09	Dominant	33.94
S5: TDF for mothers with HBeAg( +)	29.0771	0.0069	29.0408	0.0171	36.02	−4.90	Dominant	70.64
S6: Current practice	29.0703	Reference	29.0238	Reference	40.92	Reference	Reference	Reference
**From societal perspective**								
S1: Universal Vaccination	29.0606	−0.0097	28.9997	−0.0241	168.20	31.76	Dominated	−124.65
S2: HBIG for infants of mothers with HBeAg( +)	29.0644	−0.0059	29.0092	−0.0146	156.03	19.59	Dominated	−75.79
S3: HBIG for infants of mothers with HBsAg( +)	29.0665	−0.0038	29.0143	−0.0095	149.67	13.22	Dominated	−49.70
S4: TDF for mothers with high viral load	29.0738	0.0035	29.0326	0.0088	126.07	−10.38	Dominant	44.22

### Parameter uncertainty analysis

Tornado diagrams (Figure S4, Figure S5) indicated that the base-case results were reconfirmed in almost all parameter variations for both perspectives. The S4 and S5 were more cost-effective than S6 when all parameters were varied, except for the efficacy of vaccine plus HBIG compared with vaccine only in infants of HBeAg(-) mothers.

**Fig. 2:**
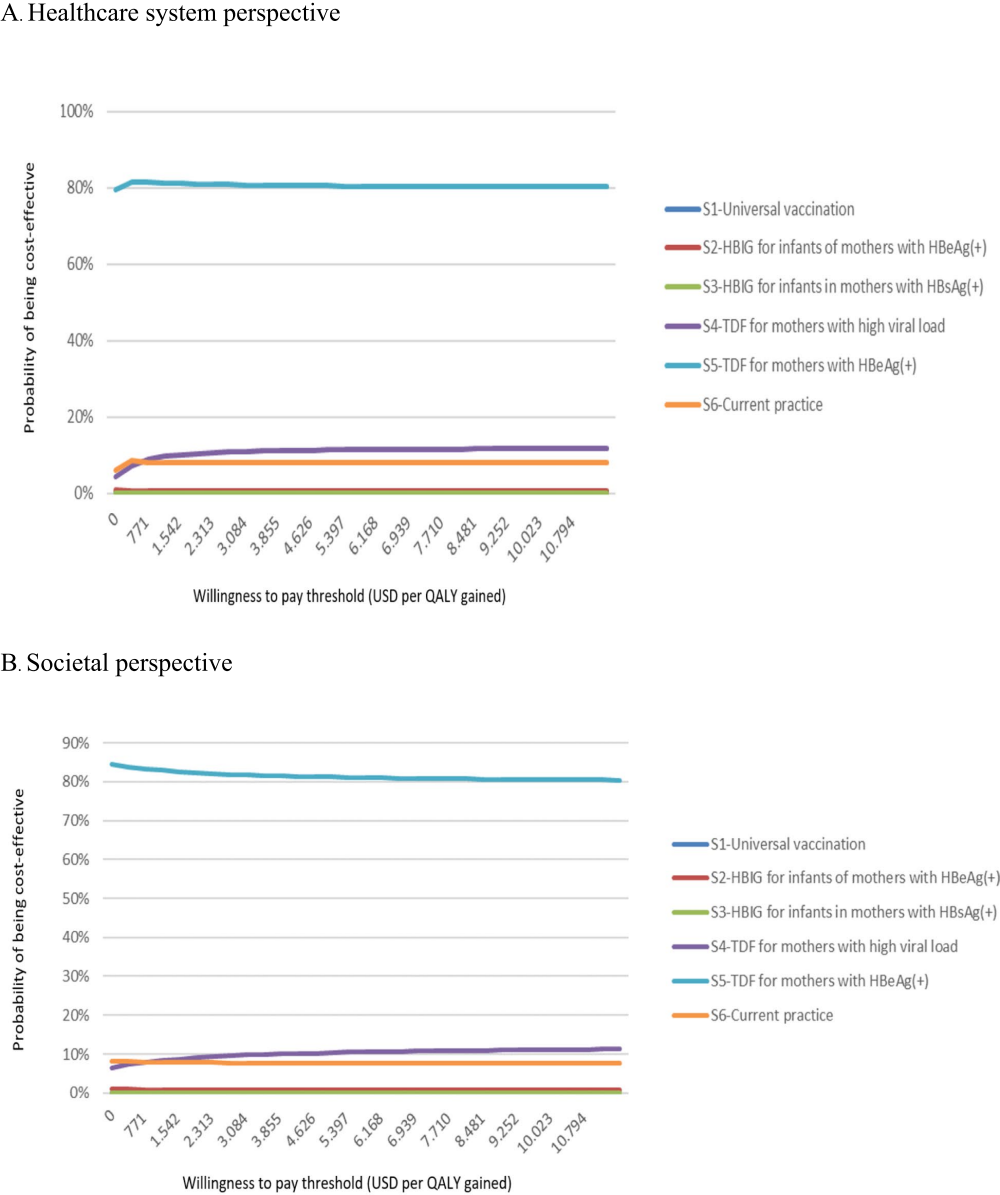
Cost-effectiveness acceptability curve by preventive strategies

Results from 10,000 simulations confirmed the robustness of the base-case analyses for both perspectives (Table S2). The S5 had the highest probability of being the most cost-effective, approximately of 80% (Fig. 2). Noticeably, the cost-effectiveness acceptability curve showed that if the S5 was excluded due to the lack of official recommendation on the TDF prophylaxis in HBeAg( +) mothers in Vietnam, the S4 had the highest probability of being the most cost-effective from both perspectives (Figure S6).

## Scenario analysis

The increase in the coverage of screening tests (i.e., HBsAg, HBeAg, HBV DNA) and HBV vaccine decreased the CHB rate among 1-year infants. It should be noted that in the most optimistic scenario with 95% of pregnant women screened for HBsAg, HBeAg or HBV DNA, and 90% of infants received HBV vaccine, strategies S4 and S5 led to lowest CHB rate of 0.3% among 1-year infants. However, this remains above the Ministry of Health and WHO target, which aims for a CHB rate of 0.1% among infants up to 5 years of age (Figure S3).

As compared to the current practice (S6), the preventive strategies in infants only (i.e., S1, S2, and S3) were not cost-effective, whereas the addition of HBIG in infants with or without the expansion of TDF prophylaxis in mothers with HBeAg( +) was cost-effective from both perspectives in all three scenarios (Table 3, Table S3). The increase of coverage of screening tests, HBV vaccine and TDF prophylaxis increased the benefit of the current practice over strategies with interventions in infants only, but also reduced the benefit of strategies S4 and S5 over the current practice.

**Table 3:** Results from scenario analyses based on healthcare system perspective

Compared strategies	Inc. QALY	Inc. Cost ($)	INMB ($)
**Base-case analysis**			
S1 vs S6	−0.0241	7.9297	−100.8149
S2 vs S6	−0.0146	6.4762	−62.6727
S3 vs S6	−0.0095	6.5359	−43.0071
S4 vs S6	0.0088	−0.0893	33.9386
S5 vs S6	0.0171	−4.8993	70.6413
Scenario 1 (50%−70%−50%−80%)*			
S1 vs S6	−0.0188	6.1868	−78.7696
S2 vs S6	−0.0114	5.0248	−48.9784
S3 vs S6	−0.0074	5.0765	−33.6379
S4 vs S6	0.0066	0.0221	25.5538
S5 vs S6	0.0121	−3.3302	50.0071
Scenario 2 (70%−90%−70%−85%)*			
S1 vs S6	−0.0339	11.3866	−142.0725
S2 vs S6	−0.0197	8.5258	−84.4584
S3 vs S6	−0.0169	9.6313	−74.8353
S4 vs S6	0.0070	1.3521	25.4777
S5 vs S6	0.0135	−3.4056	55.4746
Scenario 3 (95%−95%−95%−90%)*			
S1 vs S6	−0.0569	19.1916	−238.5717
S2 vs S6	−0.0354	14.5463	−150.8761
S3 vs S6	−0.0325	16.5366	−141.8209
S4 vs S6	0.0047	4.2853	14.0172
S5 vs S6	0.0043	0.8623	15.8426

^*^Coverage of HBsAg, HBeAg, HBV DNA and HBV vaccine birth dose, respectively

S1- Universal vaccination; S2-HBIG for infants of mothers with HBeAg( +); S3-HBIG for infants of mothers with HBsAg( +); S4-TDF for mothers with high viral load; S5-TDF for mothers with HBeAg( +); S6-Current practice; Inc.: Incremental; INMB: Incremental net monetary benefit; QALY: Quality-adjusted life year

## Discussion

The base-case analysis indicated that maternal TDF prophylaxis either in high VL (S4) or HBeAg( +) (S5) mothers plus HBIG in infants of HBsAg( +) mothers was more effective but less costly than the current practice. Similar to previous EEs from the UMIC and HIC [30–34], our study indicated that from the point of view of an LMIC, although the cost of HBV DNA quantitative test was still high, with the low cost of generic TDF and the significant cost savings from successful prevention of CHB in newborns, the addition of maternal TDF prophylaxis were still more effective and less costly than interventions in infants only.

Our study indicated that as compared to the maternal TDF prophylaxis plus HBV vaccine in infants, the addition of HBIG in infants of HBsAg( +) mothers was cost-effective, which was different from the findings by Bierhoff M. et al. [30]. The authors applied the decision tree model which estimated only screening and preventive cost but not CHB treatment cost [30]. It should be noted that from our findings, treatment cost of CHB accounted for major proportion of total lifetime costs. Without considerations of CHB treatment cost, Bierhoff et al. would lead to underestimate the benefit of addition of HBIG. For mothers with HBeAg(-) status, the risk of MTCT of HBV is minimal, especially when universal infant vaccination programs are implemented. The 2020 WHO guidelines emphasize that vaccination alone is sufficient to effectively manage MTCT risks in these cases, without requiring the administration of HBIG [13]. Therefore, HBIG is not considered beneficial for infants born to HBeAg(-) mothers. Additionally, universal vaccination also addresses horizontal transmission risks, further reducing the need for additional interventions like HBIG in these scenarios.

As proven in our study, the addition of HBIG in infants was dominant to the current practice from both healthcare and societal perspectives. However, HBIG has not yet been included in the benefit package in Vietnam. Recent updates in the 2024 WHO guidelines have revised the role of HBIG in preventing MTCT of hepatitis B [20]. While the 2020 WHO guidelines [13] recommended HBIG where available, particularly in combination with maternal TDF prophylaxis and universal infant vaccination, the 2024 guidelines no longer recommend the routine use of HBIG, especially in LMIC [20]. HBIG is now primarily suggested in high-resource settings for infants born to HBsAg-positive mothers with high viral loads. This change reflected the limited data supporting the additional benefits of HBIG when used alongside TDF and universal vaccination. Supporting this, a study conducted in the Democratic Republic of the Congo involving nine women found high protective efficacy of maternal TDF combined with infant hepatitis B vaccination, even without HBIG administration [35]. This evidence suggests that maternal TDF prophylaxis alone may be sufficient for effective prevention in many settings, though further large-scale studies are needed to confirm these findings. Consistent with these guidelines, our study demonstrates that incorporating HBIG for infants born to HBsAg-positive mothers, alongside expanding TDF prophylaxis to HBeAg-positive mothers, remains highly cost-effective or even dominant compared to current practices. Similar to previous EEs [30, 36, 37], our study indicated that the HBeAg test, used as a proxy of high VL was dominant to HBV DNA quantitative test due to the HBeAg test’s broader coverage and lower cost. These findings align with WHO recommendations, which suggest the use of the HBeAg test, especially in regions where HBV DNA quantitative tests are not available. Our study could provide additional evidence on cost-effectiveness to support this recommendation.

To achieve the goal of HBV elimination, one important strategy is to reduce the prevalence of HBsAg( +) in children under 5 years to 0.1% or less [5, 29]. However, our study indicated that among 1-year-old children, the incidence of CHB in the current practice was 0.97% and reduced to 0.76% in strategy S5. At the most promising scenario, i.e., 95% of pregnant women were screened for HBsAg, HBeAg or HBV DNA, and 90% of infants received vaccine, incidence of CHB in strategies S4 and S5 reduced to 0.3% among one-year-old infants, which were still higher than the WHO’s target of 0.1% for children up to five years. Our results indicated that focusing on vertical transmission was very important, however, this seemed to be not enough to eliminate HBV in Vietnam. Other strategies to screen for HBsAg in general population and provide appropriate interventions need to be taken into account. To comprehensively estimate the benefit of alternative prevention strategies, future studies could consider both vertical and horizontal transmission as well as estimate the benefit from maternal side.

Additionally, the findings highlight the necessity of enhancing the quality and accessibility of universal immunization and screening programs. This could enable earlier identification of high-risk pregnancies and optimize the timing and effectiveness of immunization for infants. To achieve this, increased investment from the Vietnamese government is crucial, particularly in health infrastructure, healthcare worker training, and community outreach efforts. These improvements would ensure more comprehensive and timely screening and vaccination coverage. Furthermore, the WHO Triple Elimination Initiative seeks to eradicate MTCT of human immunodeficiency viruses (HIV), syphilis, and hepatitis B simultaneously [38]. The use of a multiplex test, which integrates screening efforts for these infections, could yield cost savings by combining tests and lead to improved health outcomes for both mothers and infants [38]. The WHO Triple Elimination Initiative emphasizes the importance of strategic integration in screening practices, which should be a focal point for Vietnamese policymakers when planning future health programs.

As for our knowledge, our study is the first EE comparing long-term outcomes and costs that was performed in an LMIC with high prevalence of HBV taking into account the WHO recent recommendation on the usage of HBeAg test as a proxy of high VL. Our study could provide the evidence to support the considerations of the most cost-effective alternatives to prevent MTCT of HBV – an important task toward HBV elimination in Vietnam or other countries with similar contexts. A hybrid model was used to estimate both short-term and long-term costs and health outcomes of six preventive strategies, which was suitable for a life-long disease, especially when serious consequences tended to occur at the older age. Clinical experts were involved in model structure adjustment and assumptions to ensure that patients pathway reflected the local context.

Nevertheless, limitations in the study should be addressed. First, the model ignored horizontal transmission and assumed the lifelong protection of 4-dose schedule of vaccine. It should be noted that WHO did not recommend booster vaccination for people who did complete three doses of HBV vaccination due to the prolonged protection [1]. Therefore, horizontal transmission mostly happened to infants who were not vaccinated. In this study, we applied the same coverage of HBV vaccine in all six compared strategies, therefore the impact of horizontal transmission on all strategies was similar and the ignorance of this route of transmission seemed to not significantly impact the main results. Second, due to the lack of data in Vietnam, some parameters were obtained from literatures in other countries. However, several approaches were applied to overcome this limitation, including the prioritization of parameters from high level of evidence, consultation with local experts and performance of uncertainty analyses.

Our results suggested that the current practice was dominant to all strategies with interventions in infants only. In contrast, the addition of HBIG in infants of HBsAg( +) mothers with/without the expansion of TDF prophylaxis to HBeAg( +) mothers was very cost-effective or even dominant to the current practice. The results from this study could provide very useful evidence to support policy decision making in Vietnam, as well as other LMIC or LIC with similar context, especially in countries with high prevalence of HBsAg and aiming towards HBV elimination.

## Supplementary Information

Supplementary Material 1.

Supplementary Material 2.

## Data Availability

All data generated or analysed during this study are included in this published article and its supplementary information files.

## Abbreviations

MTCT: Mother-to-child transmission
HBB: Hepatitis B virus
HBIG: Hepatitis B immunoglobulin
TDF: Tenofovir disoproxil fumarate
VL: Viral load
WHO: World Health Organization
BD: Birth dose
NEPI: National Expand Program on Immunization
UMIC: Upper-middle-income country
LMIC: Lower-middle-income country
LBIT: Laboratory based immunoassay test
CHB: Chronic hepatitis B
CC: Compensated cirrhosis
DCC: Decompensated cirrhosis
HCC: Hepatocellular carcinoma
RDT: Rapid diagnostic test
RCT: Randomized controlled trials
DMC: Direct medical cost
EE: Economic evaluation
WTP: Willingness-to-pay
INMB: Incremental net monetary benefits
QALY: Quality adjusted life years
SE: Standard error
CI: Confidence interval
PSA: Probabilistic sensitivity analysis
