# Supplementary material for: Economic evaluation of tenofovir disoproxil fumarate prophylaxis to prevent mother-to-child transmission of Hepatitis B virus infection: evidence from a lower-middle income country
Source: BMC Health Serv Res. 2024 Dec 28;24:1658. doi: 10.1186/s12913-024-12152-z (PMC11681667; doi:10.1186/s12913-024-12152-z)
Supplement: Supplementary file 1 — Supplementary Material 1. [file 12913_2024_12152_MOESM1_ESM.docx]

# Appendix S1. Additional figures

Figure S1. Decision tree model structure

Figure S2. Cost components in base-case of six strategies

## Figure S3. Incidence of HBV infection among 1-year-old infants

Figure S4. Tornado diagram from healthcare system perspective

Figure S5. Tornado diagram from societal perspective

Figure S6. Cost-effectiveness acceptability curve without strategy S5 (TDF for mothers with HBeAg(+))


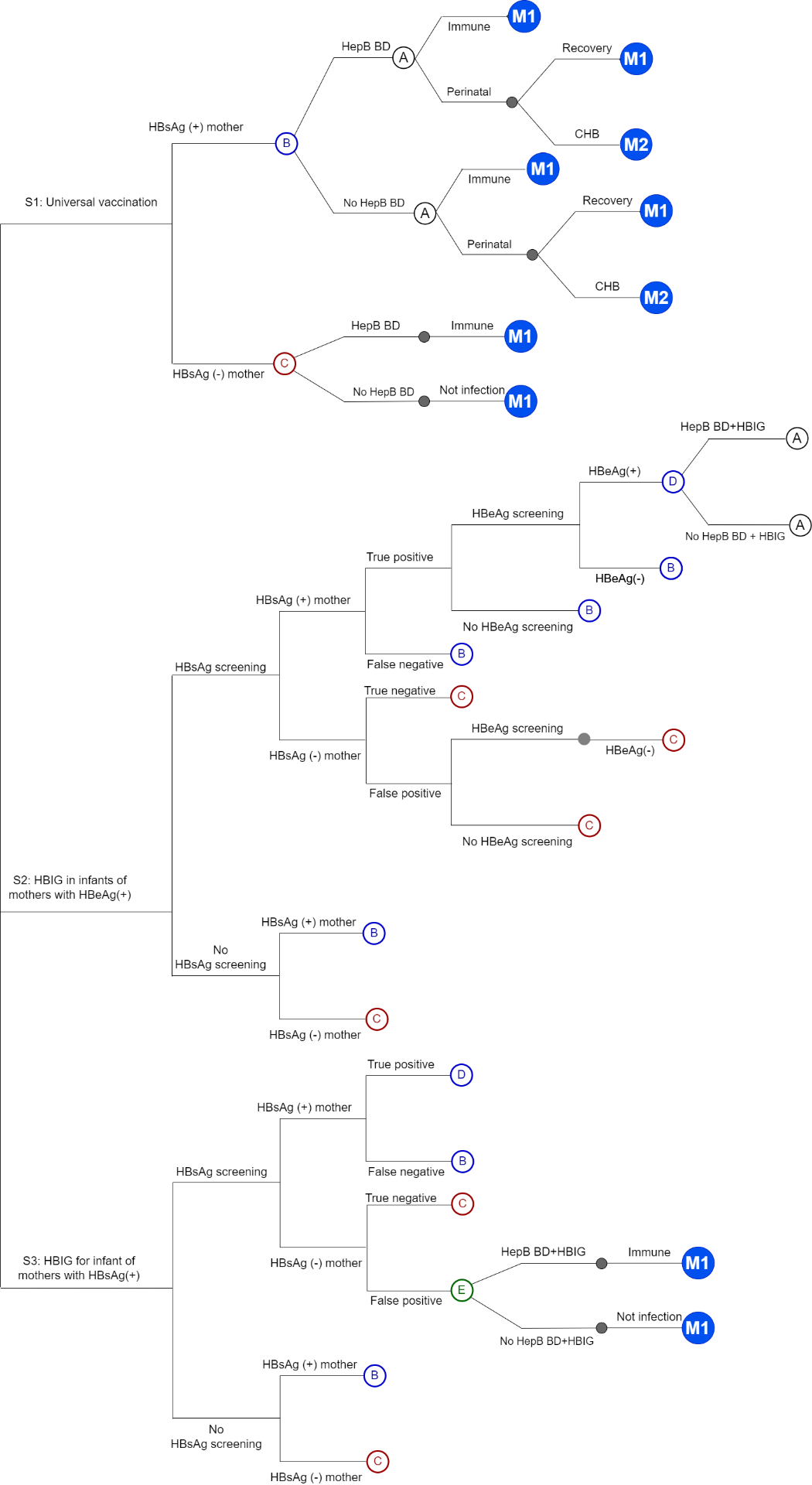


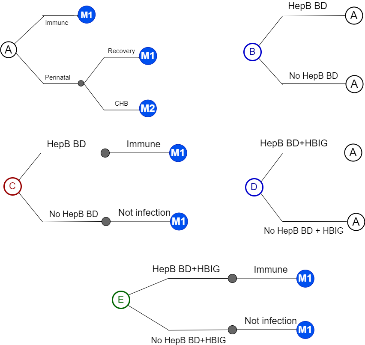


In which:

## Figure S1. Decision tree model structure


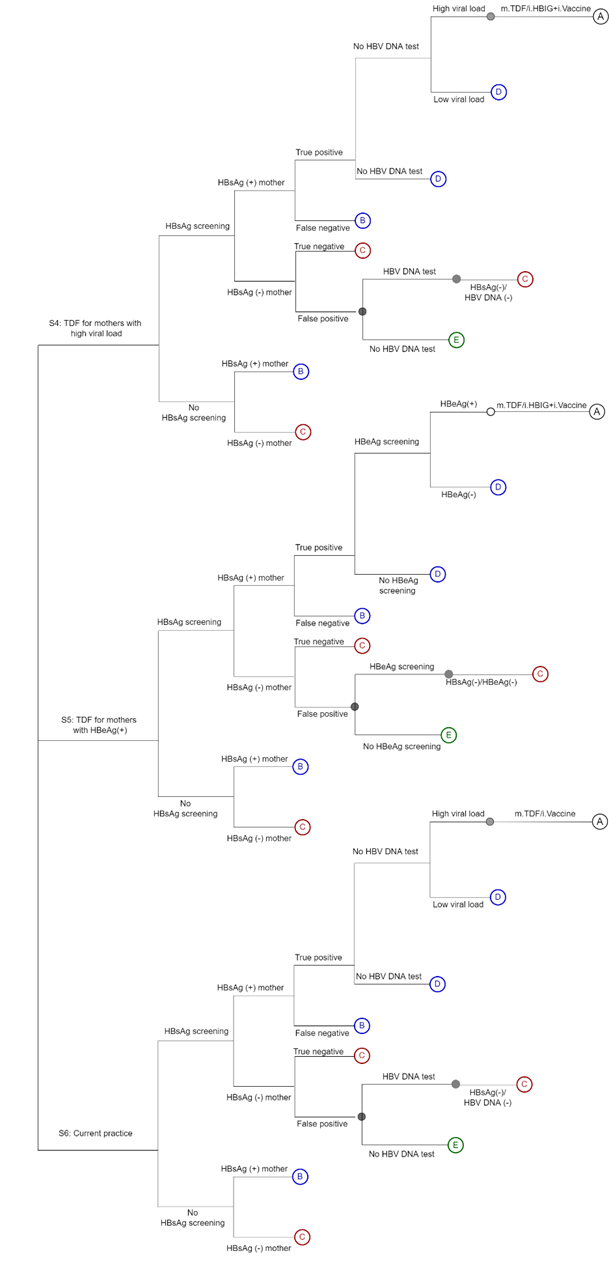


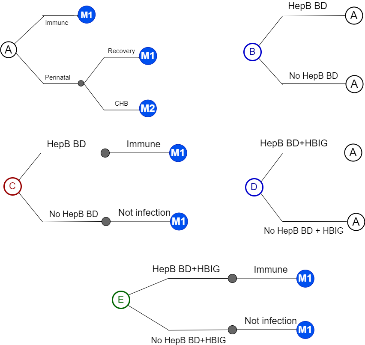


In which:

Figure S1. Decision tree model structure (Cont.)

A. Healthcare system perspective

*
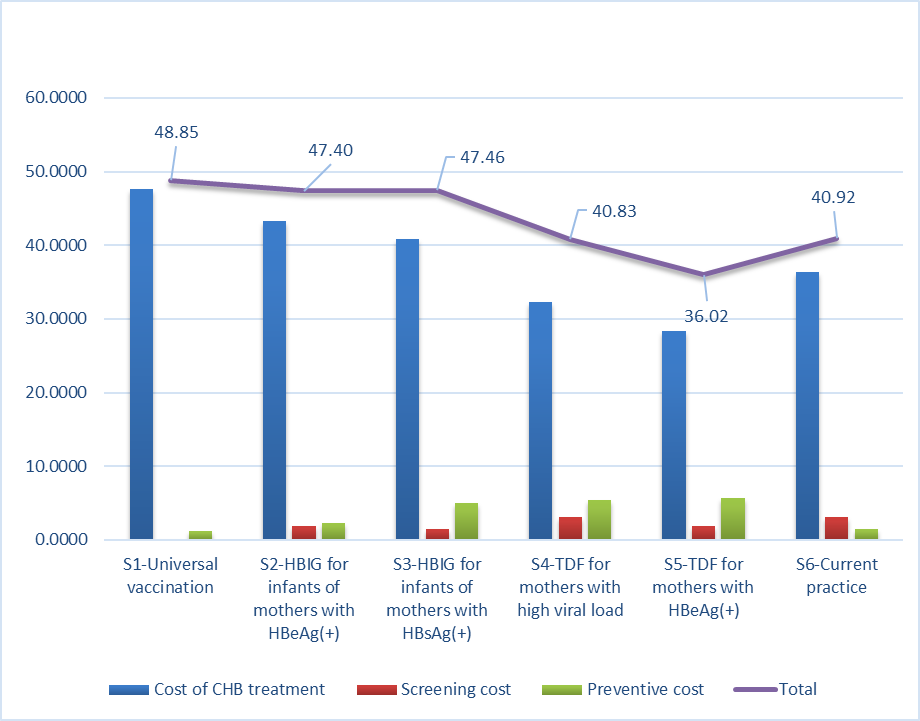
*


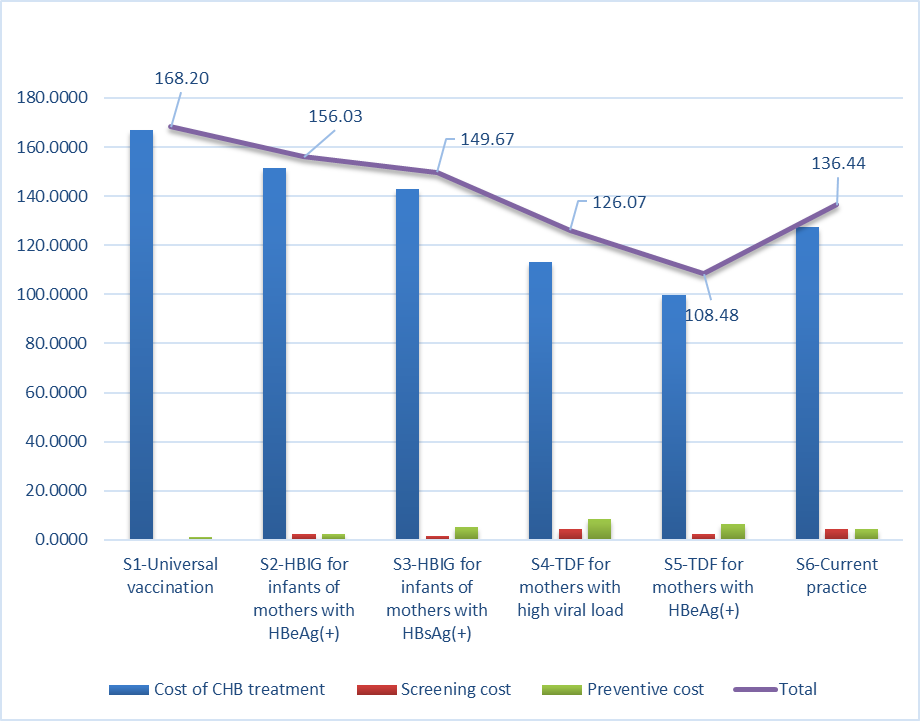
B. Societal perspective

## Figure S2. Cost components in base-case of six strategies


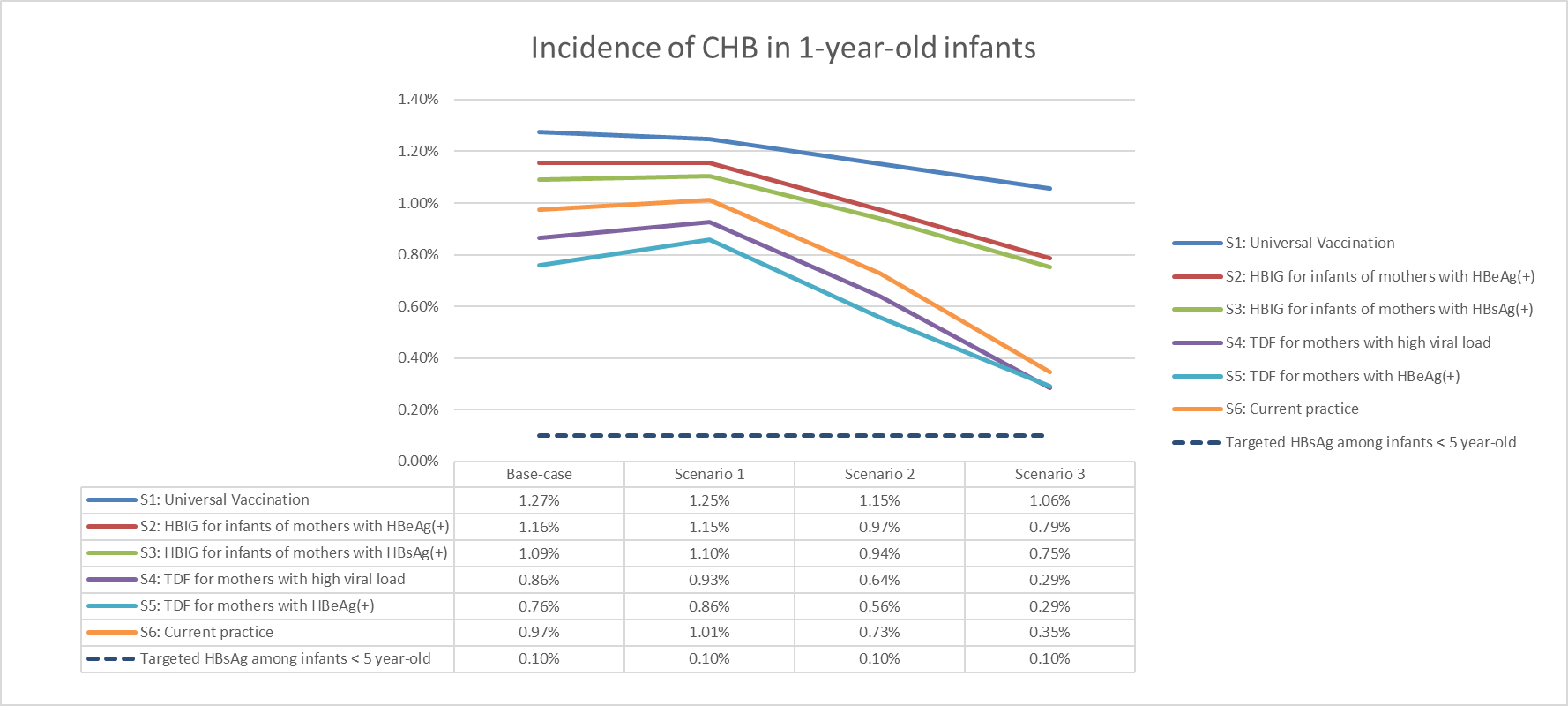


*Coverage of HBsAg, HBeAg, HBV DNA and HBV vaccine birth dose in different scenarios: Base case: 65.30% - 70% - 47.83% - 78.5%; Scenario 1: 50% - 70% - 50% -80%; Scenario 2: 70% - 90% - 70% - 85%; Scenario 3: 95% - 95% - 95% - 90%*

## Figure S3. Incidence of HBV infection among 1-year-old infants


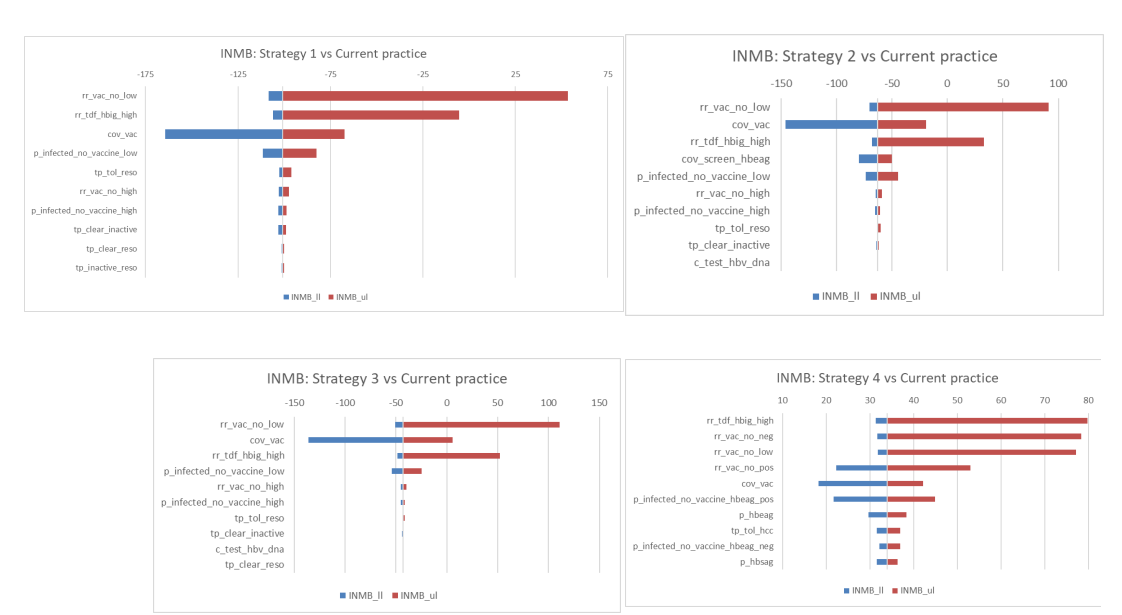

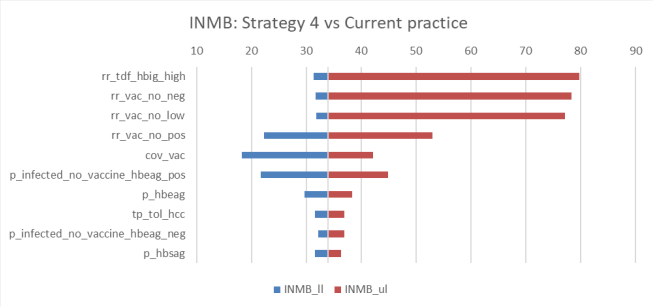


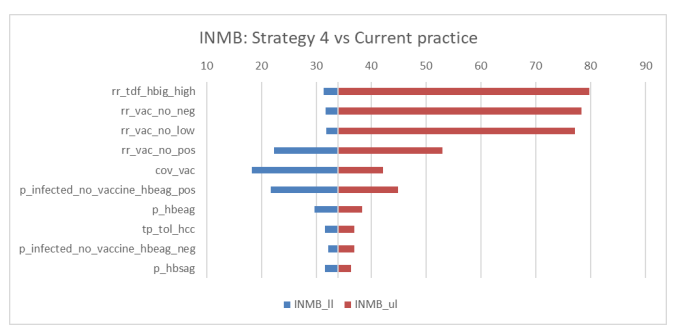


## Figure S4. Tornado diagram from healthcare system perspective


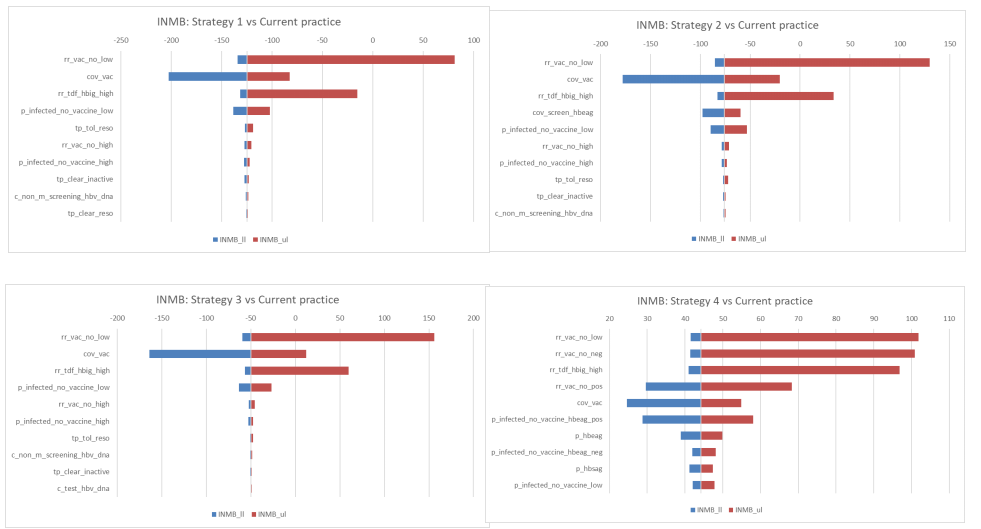

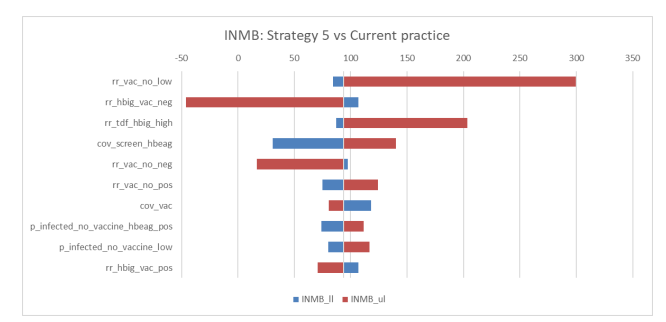


## Figure S5. Tornado diagram from societal perspective

A. Healthcare system perspective


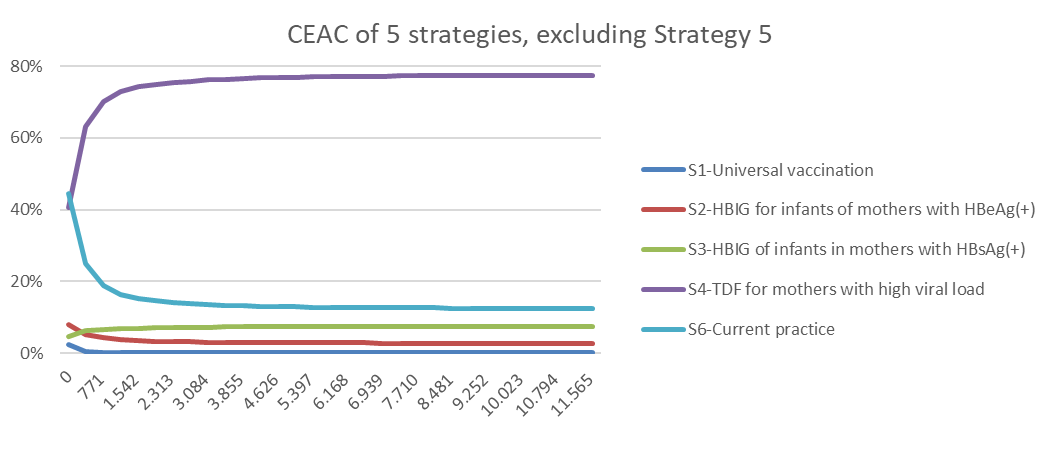


B. Societal perspective


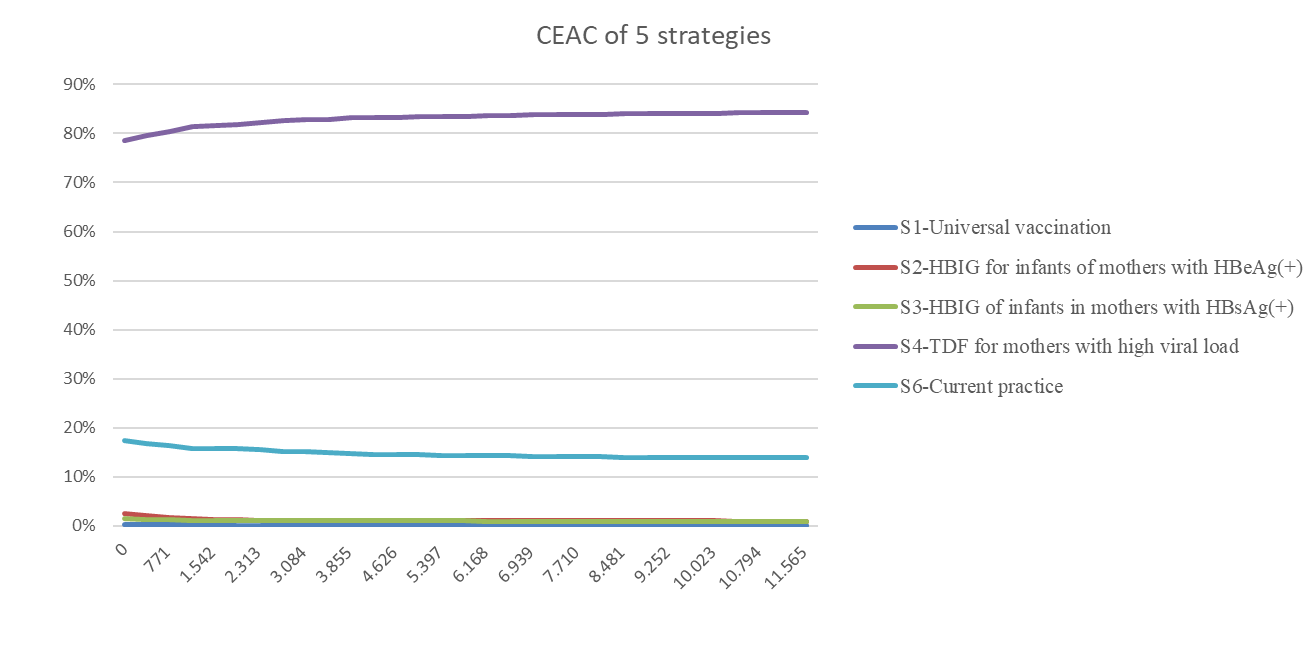


## Figure S6. Cost-effectiveness acceptability curve without strategy S5 (TDF for mothers with HBeAg(+))
