# Supplementary material for: Economic evaluation of tenofovir disoproxil fumarate prophylaxis to prevent mother-to-child transmission of Hepatitis B virus infection: evidence from a lower-middle income country
Source: BMC Health Serv Res. 2024 Dec 28;24:1658. doi: 10.1186/s12913-024-12152-z (PMC11681667; doi:10.1186/s12913-024-12152-z)
Supplement: Supplementary file 2 — Supplementary Material 2. [file 12913_2024_12152_MOESM2_ESM.docx]

# Appendix S2. Additional tables

Table S1. Input parameters used in the model

Table S2. Incremental net monetary benefit of compared strategies (PSA)

Table S3. Results from scenario analysis (From societal perspective)

**List of abbreviation**

CHB: Chronic hepatitis B

CC: Compensated cirrhosis

DCC: Decompensated cirrhosis

HBIG: Hepatitis B immunoglobulin

HCC: Hepatocellular carcinoma

MTCT: Mother-to-child transmission

NA: Not applicable

RDT: Rapid diagnostic test

SE: Standard error

TDF: Tenofovir disoproxil fumarate

## Table S1. Input parameters used in the model

| **Input parameters** | **Mean** | **SE** | **Distribution** | **Source** | |
| --- | --- | --- | --- | --- | --- |
| **Epidemiology** | | | | |  |
| 2022 birth cohort | 1,568,163 | NA | NA | GSO’s data | |
| Prevalence of HBsAg among pregnant women | 10.83% | 0.38% | Beta | ^1^ | |
| Proportion of HBeAg(+) among mothers with HBsAg(+) | 42.06% | 3.23% | Beta | ^2^ | |
| Proportion of high viral load among mothers with HBsAg(+) | 40.16% | 4.44% | Beta | ^2^ | |
| **Screening effectiveness** | | | | |  |
| Coverage of HBsAg | 65.30% | 0.038% | Beta | Department of Maternal Health and children | |
| Coverage of HBV DNA | 47.83% | 2.69% | Beta | ^3^ | |
| Coverage of HBeAg | 70.00% | 14.00% | Beta | Expert consultation | |
| Sensitivity of HBsAg RDT | 90.00% | 0.43% | Beta | ^4^ | |
| Specificity of HBsAg RDT | 99.50% | 0.03% | Beta | ^4^ | |
| **Efficacy and coverage of interventions** | | | | |  |
| Coverage of HBV vaccine birth dose | 78.50% | 15.70% | Beta | ^5^ | |
| Percentage of perinatal infection in infants without any interventions | | | | |  |
| In mothers with HBsAg(+)/HBeAg(+) or high viral load | 61.48% | 12.03% | Beta | Meta-analysis ^6, 7^ | |
| In mothers with HBsAg(+)/HBeAg(-) or low viral load | 5.03% | 2.73% |  | Meta-analysis ^8-10^ | |
| Risk ratios on MTCT of HBV | | | | |  |
| Mothers with HBeAg(+) |  |  |  |  | |
| Vaccine vs no intervention | 0.31 | 0.24 | Log-normal | Network meta-analysis | |
| HBIG and vaccine vs vaccine | 0.52 | 0.28 | Log-normal | Network meta-analysis | |
| Maternal TDF vs no TDF | 0.10 | 0.63 | Log-normal | Network meta-analysis | |
| Mothers with high viral load |  |  |  |  | |
| Vaccine vs no intervention | 0.31 | 0.24 | Log-normal | Assumption | |
| HBIG and vaccine vs vaccine | 0.52 | 0.28 | Log-normal | Assumption | |
| Maternal TDF vs no TDF | 0.08 | 1.46 | Log-normal | ^11^ | |
| Mothers with HBeAg(-) or low viral load |  |  |  |  | |
| Vaccine vs no intervention | 0.36 | 1.53 | Log-normal | ^7^ | |
| HBIG and vaccine vs vaccine | 0.72 | 1.20 | Log-normal | Meta-analysis ^12, 13^ | |

Table S1. Input parameters used in the model (Cont.)

| **Input parameters** | **Mean** | **SE** | **Distribution** | **Source** |
| --- | --- | --- | --- | --- |
| **Transition probabilities** | | | | |
| From perinatal transmission to immune tolerant phase | 88.50% | 0.02% | Beta | ^14^ |
| From immune tolerance to |  |  |  |  |
| HBeAg(+) CHB |  |  |  |  |
| < 6 years old | 0.50% | 0.28% | Beta | ^15^ |
| 6-12 years old | 1.95% | 0.63% | Beta | ^15^ |
| > 12 years old | 5.12% | 1.00% | Beta | ^15^ |
| Resolution | 0.12% | 0.12% | Beta | ^16^ |
| HCC | 0.36% | 0.11% | Beta | ^17^ |
| From HBeAg(+) CHB to |  |  |  |  |
| Inactive CHB | 8.53% | 1.71% | Beta | ^18^ |
| Resolution | 0.27% | 0.10% | Beta | ^19^ |
| CC | 1.75% | 0.17% | Beta | ^20^ |
| HCC | 0.49% | 0.09% | Beta | ^21^ |
| From inactive CHB to |  |  |  |  |
| HBeAg(+) CHB | 0.50% | 0.14% | Beta | ^22^ |
| HBeAg(-) CHB | 3.14% | 0.30% | Beta | ^22^ |
| Resolution | 0.82% | 0.14% | Beta | ^19^ |
| CC | 0.06% | 0.06% | Beta | ^22^ |
| HCC | 0.19% | 0.11% | Beta | ^22^ |
| From HBeAg(-) CHB to |  |  |  |  |
| Resolution | 0.97% | 0.28% | Beta | ^19^ |
| CC | 2.52% | 0.54% | Beta | ^22^ |
| HCC | 0.49% | 0.09% | Beta | ^21^ |
| From CC to |  |  |  |  |
| DCC | 3.11% | 0.61% | Beta | ^23^ |
| HCC | 3.31% | 0.45% | Beta | ^21^ |
| Death | 5.26% | 0.89% | Beta | ^24^ |
| From DCC |  |  |  |  |
| HCC | 7.89% | 1.28% | Beta | ^25, 26^ |
| Death | 18.29% | 1.39% | Beta | ^24^ |
| From HCC to death | 47.88% | 2.27% | Beta | ^27^ |

Table S1. Input parameters used in the model (Cont.)

| **Input parameters** | **Mean** | **SE** | **Distribution** | **Source** |
| --- | --- | --- | --- | --- |
| **Costs** | | | | |
| ***Cost of screening tests and prophylactic interventions*** |  |  |  |  |
| HBsAg RDT* | 2.37 | 0.47 | Gamma | National list for medical service |
| HBsAg laboratory-based test* | 3.31 | 0.66 | Gamma |  |
| HBeAg laboratory-based test* | 4.23 | 0.85 | Gamma |  |
| HBV DNA quantitative test* | 44.11 | 8.82 | Gamma |  |
| HBV vaccine birth dose | 0.56 | 0.11 | Gamma | ^28^ |
| HBIG (180 IU/ml) | 74.74 | 14.95 | Gamma | Bidding results |
| Tenofovir disoproxil fumarate (300 mg/tablet) | 0.14 | 0.03 | Gamma | Bidding results |
| Implementation cost of HBV vaccine birth dose | 0.93 | 0.19 | Gamma | ^29^ |
| Direct non-medical cost of HBeAg test | 2.07 | 0.41 | Gamma | ^30^, Expert consultation |
| Direct non-medical cost of HBV DNA quantitative test | 32.80 | 6.56 | Gamma | ^31^, Expert consultation |
| Time cost of HBeAg test | 7.23 | 1.45 | Gamma | Assumption |
| Time cost of HBV DNA test | 14.46 | 2.90 | Gamma | Assumption |
| ***Health state costs*** |  |  |  |  |
| Direct medical cost (including both payment by public health insurance and patients)* | | | | |
| Immune tolerance | 94.41 | 6.99 | Gamma | Primary data in Vietnam |
| HBeAg(+) CHB | 133.74 | 7.04 | Gamma | Primary data in Vietnam |
| Inactive CHB | 105.19 | 4.53 | Gamma | Primary data in Vietnam |
| HBeAg(-) CHB | 179.13 | 12.45 | Gamma | Primary data in Vietnam |
| CC | 652.05 | 130.41 | Gamma | ^31^ |
| DCC | 1049.96 | 209.99 | Gamma | ^31^ |
| HCC | 4003.22 | 800.64 | Gamma | ^31^ |
| Direct non-medical cost (Transportation, meal, and accommodation) | | | | |
| Immune tolerance | 188.86 | 37.75 | Gamma | ^31^ |
| HBeAg(+) CHB | 188.86 | 37.75 | Gamma |  |
| Inactive CHB | 188.86 | 37.75 | Gamma |  |
| HBeAg(-) CHB | 188.86 | 37.75 | Gamma |  |
| CC | 230.19 | 45.99 | Gamma |  |
| DCC | 395.40 | 79.08 | Gamma |  |
| HCC | 362.53 | 72.46 | Gamma |  |

**Costs for medical services announced in the National list that included 4 components: personnel, drug/material/supplies, operations, maintenance*

Table S1. Input parameters used in the model (Cont.)

| **Input parameters** | **Mean** | **SE** | **Distribution** | **Source** |
| --- | --- | --- | --- | --- |
| **Costs** | | | | |
| ***Health state costs*** |  |  |  |  |
| Time cost (Time cost of patients and informal caregivers) |  |  |  |  |
| Immune tolerance | 205.89 | 41.22 | Gamma | ^31^ |
| HBeAg(+) CHB | 205.89 | 41.22 | Gamma |  |
| Inactive CHB | 205.89 | 41.22 | Gamma |  |
| HBeAg(-) CHB | 205.89 | 41.22 | Gamma |  |
| CC | 218.69 | 43.72 | Gamma |  |
| DCC | 401.26 | 80.27 | Gamma |  |
| HCC | 392.15 | 78.43 | Gamma |  |
| **Utility** |  |  |  |  |
| No HBV infection/Resolution | 1 | NA | NA | Assumption |
| Immune tolerance | 0.81 | 0.003 | Beta | ^32^ |
| HBeAg(+) CHB | 0.77 | 0.007 | Beta | ^32^ |
| Inactive CHB | 0.81 | 0.004 | Beta | ^32^ |
| HBeAg(-) CHB | 0.77 | 0.01 | Beta | ^32^ |
| CC | 0.70 | 0.08 | Beta | ^31^ |
| DCC | 0.49 | 0.16 | Beta | ^31^ |
| HCC | 0.36 | 0.16 | Beta | ^31^ |

*SE: Standard error; RDT: Rapid diagnostic test; MTCT: Mother-to-child transmission; CHB: Chronic hepatitis B; TDF: Tenofovir disoproxil fumarate; HBIG: Hepatitis B immunoglobulin; CC: Compensated cirrhosis; DCC: Decompensated cirrhosis; HCC: Hepatocellular carcinoma; NA: Not applicable*

Table S2. Incremental net monetary benefit of compared strategies (PSA)

| **Prophylactic strategy** | **LY** | **Inc. LY** | **QALY** | **Inc. QALY** | **Cost ($)** | **Inc. Cost ($)** | **ICER** | **INMB**  **($)** |
| --- | --- | --- | --- | --- | --- | --- | --- | --- |
| **From healthcare system perspective** | | | | | | | | |
| S1: Universal Vaccination | 29.0545 | -0.0086 | 28.9841 | -0.0216 | 56.2494 | 6.7695 | Dominated | -89.6666 |
| S2: HBIG for infants of mothers with HBeAg(+) | 29.0583 | -0.0049 | 28.9934 | -0.0123 | 54.8921 | 5.4121 | Dominated | -52.7519 |
| S3: HBIG for infants of mothers with HBsAg(+) | 29.0580 | -0.0052 | 28.9926 | -0.0130 | 57.6439 | 8.1640 | Dominated | -58.2508 |
| S4: TDF for mothers with high viral load | 29.0645 | 0.0013 | 29.0088 | 0.0032 | 51.9993 | 2.5194 | $1,927.46/LY  $794.05/QALY | 9.7115 |
| S5: TDF for mothers with HBeAg(+) | 29.0688 | 0.0057 | 29.0197 | 0.0140 | 45.9571 | -3.5229 | Dominant | 57.6563 |
| S6: Current practice | 29.0632 | Reference | 29.0056 | Reference | 49.4800 | Reference | Reference | Reference |
| **From societal perspective** | | | | | | | | |
| S1: Universal Vaccination | 29.0547 | -0.0087 | 28.9848 | -0.0217 | 193.0176 | 27.8319 | Dominated | -111.2913 |
| S2: HBIG for infants of mothers with HBeAg(+) | 29.0585 | -0.0049 | 28.9942 | -0.0123 | 181.0886 | 15.9030 | Dominated | -63.2819 |
| S3: HBIG for infants of mothers with HBsAg(+) | 29.0575 | -0.0060 | 28.9916 | -0.0149 | 188.0173 | 22.8316 | Dominated | -80.0845 |
| S4: TDF for mothers with high viral load | 29.0642 | 0.0008 | 29.0085 | 0.0021 | 166.3199 | 1.1343 | $1,399.51/LY  $551.26/QALY | 6.7976 |
| S5: TDF for mothers with HBeAg(+) | 29.0682 | 0.0048 | 29.0184 | 0.0119 | 146.3676 | -18.8180 | Dominant | 64.7609 |
| S6: Current practice | 29.0634 | Reference | 29.0065 | Reference | 165.1856 | Reference | Reference | Reference |

*LY: life years; QALY: Quality-Adjusted life years; Inc.: Incremental; ICER: Incremental Cost-Effectiveness Ratio; INMB: Incremental Net Monetary Benefit*

## Table S3. Results from scenario analysis (From societal perspective)

| **Compared strategies** | **Inc. QALY** | **Inc. Cost ($)** | **INMB ($)** |
| --- | --- | --- | --- |
| **Base-case analysis** | | | |
| S1 vs S6 | -0.0241 | 31.7609 | -124.6462 |
| S2 vs S6 | -0.0146 | 19.5921 | -75.7887 |
| S3 vs S6 | -0.0095 | 13.2248 | -49.6960 |
| S4 vs S6 | 0.0088 | -10.3750 | 44.2243 |
| S5 vs S6 | 0.0171 | -27.9654 | 93.7073 |
| **Scenario 1 (50%-70%-50%-80%)*** | | | |
| S1 vs S6 | -0.0188 | 24.7253 | -97.3081 |
| S2 vs S6 | -0.0114 | 15.1955 | -59.1491 |
| S3 vs S6 | -0.0074 | 10.2383 | -38.7997 |
| S4 vs S6 | 0.0066 | -7.7496 | 33.3254 |
| S5 vs S6 | 0.0121 | -20.0321 | 66.7091 |
| **Scenario 2 (70%-90%-70%-85%)*** | | | |
| S1 vs S6 | -0.0339 | 44.2044 | -174.8903 |
| S2 vs S6 | -0.0197 | 25.3029 | -101.2354 |
| S3 vs S6 | -0.0169 | 22.5512 | -87.7552 |
| S4 vs S6 | 0.0070 | -6.8006 | 33.6303 |
| S5 vs S6 | 0.0135 | -24.3234 | 76.3924 |
| **Scenario 3 (95%-95%-95%-90%)*** | | | |
| S1 vs S6 | -0.0569 | 73.1575 | -292.5377 |
| S2 vs S6 | -0.0354 | 44.1312 | -180.4611 |
| S3 vs S6 | -0.0325 | 41.9099 | -167.1942 |
| S4 vs S6 | 0.0047 | -1.2762 | 19.5787 |
| S5 vs S6 | 0.0043 | -14.3351 | 31.0400 |

**Coverage of HBsAg, HBeAg, HBV DNA and HBV vaccine birth dose, respectively.*

*S1- Universal vaccination; S2-HBIG for infants of mothers with HBeAg(+); S3-HBIG for infants of mothers with HBsAg(+); S4-TDF for mothers with high viral load; S5-TDF for mothers with HBeAg(+); S6-Current practice; Inc.: Incremental; INMB: Incremental net monetary benefit*

**Reference**

1. Flower B, Du Hong D, Vu Thi Kim H, et al. Seroprevalence of Hepatitis B, C and D in Vietnam: A systematic review and meta-analysis. Lancet Reg Health West Pac 2022;24.

2. Miyakawa M, Yoshida LM, Nguyen HT, et al. Hepatitis B virus infection among pregnant mothers and children after the introduction of the universal vaccination program in Central Vietnam. Sci Rep 2021;11(1):8676.

3. Pham TND, Le DH, Dao DVB, et al. Establishing baseline framework for hepatitis B virus micro-elimination in Ho Chi Minh City, Vietnam - A community-based seroprevalence study. The Lancet Regional Health – Western Pacific 2022;30:100620.

4. Amini A, Varsaneux O, Kelly H, et al. Diagnostic accuracy of tests to detect hepatitis B surface antigen: a systematic review of the literature and meta-analysis. BMC Infect Dis 2017;17(Suppl 1):698.

5. General Statistics Office. Viet Nam Multiple Indicator Cluster Survey (MICS) 2014; 2014: 77-80.

6. Farmer K, Gunn T, Woodfield DG. Passive immunoprophylaxis of hepatitis B virus infections in newborn infants. N Z Med J 1985;98(788):851-3.

7. Xu ZY, Francis DP, Liu CB, et al. Prevention of hepatitis B virus carriage of infants using HBV vaccine in Shanghai. Preliminary report of a randomized double-blind placebo-controlled trial. Chin Med J (Engl) 1985;98(9):623-6.

8. Beasley RP, Hwang LY, Stevens CE, et al. Efficacy of hepatitis B immune globulin for prevention of perinatal transmission of the hepatitis B virus carrier state: final report of a randomized double-blind, placebo-controlled trial. Hepatology 1983;3(2):135-41.

9. Pojanagaroon B, Boonmar S, Chatiyanonda K, et al. Protective efficacy of plasma-derived hepatitis beta vaccine in preventing perinatal transmission of HBV infection in infants of HBsAg/HBeAg positive mothers. Southeast Asian J Trop Med Public Health 1988;19(4):615-21.

10. Wong VW, Reesink H, Ip HH, et al. Prevention of the HBsAg carrier state in newborn infants of mothers who are chronic carriers of HBsAg and HBeAg by administration of hepatitis B vaccine and Hepatitis B immunoglobulin. Double-blind Randomised Placebo-controlled study. Lancet 1984;323(8383):921-6.

11. Pan CQ, Duan Z, Dai E, et al. Tenofovir to Prevent Hepatitis B Transmission in Mothers with High Viral Load. N Engl J Med 2016;374(24):2324-34.

12. Xu ZY, Duan SC, Margolis HS, et al. Long-term efficacy of active postexposure immunization of infants for prevention of hepatitis b virus infection. J Infect Dis 1995;171(1):54-60.

13. Yang YJ, Liu CC, Chen TJ, et al. Role of hepatitis B immunoglobulin in infants born to hepatitis B e antigen-negative carrier mothers in Taiwan. Pediatr Infect Dis J 2003;22(7):584‐8.

14. Edmunds WJ, Medley GF, Nokes DJ, Hall AJ, Whittle HC. The influence of age on the development of the hepatitis B carrier state. Proceedings Biological sciences 1993;253(1337):197-201.

15. Hong SJ, Park HJ, Chu MA, Choi BS, Choe B-H. The Rate of Conversion from Immune-tolerant Phase to Early Immune-clearance Phase in Children with Chronic Hepatitis B Virus Infection. Pediatr Gastroenterol Hepatol Nutr 2014;17(1):41-6.

16. Komatsu H, Inui A, Sogo T, Tsunoda T, Fujisawa T. Chronic hepatitis B virus infection in children and adolescents in Japan. J Pediatr Gastroenterol Nutr 2015;60(1):99-104.

17. Lee HA, Lee HW, Kim IH, et al. Extremely low risk of hepatocellular carcinoma development in patients with chronic hepatitis B in immune-tolerant phase. Aliment Pharmacol Ther 2020;52(1):196-204.

18. Chan HL, Fung S, Seto WK, et al. Tenofovir alafenamide versus tenofovir disoproxil fumarate for the treatment of HBeAg-positive chronic hepatitis B virus infection: a randomised, double-blind, phase 3, non-inferiority trial. The lancet Gastroenterology & hepatology 2016;1(3):185-95.

19. Liaw YF, Sheen IS, Chen TJ, Chu CM, Pao CC. Incidence, determinants and significance of delayed clearance of serum HBsAg in chronic hepatitis B virus infection: a prospective study. Hepatology 1991;13(4):627-31.

20. Poh Z, Goh BB, Chang PE, Tan CK. Rates of cirrhosis and hepatocellular carcinoma in chronic hepatitis B and the role of surveillance: a 10-year follow-up of 673 patients. Eur J Gastroenterol Hepatol 2015;27(6):638-43.

21. Raffetti E, Fattovich G, Donato F. Incidence of hepatocellular carcinoma in untreated subjects with chronic hepatitis B: a systematic review and meta-analysis. Liver Int 2016;36(9):1239-51.

22. Hsu YS, Chien RN, Yeh CT, et al. Long-term outcome after spontaneous HBeAg seroconversion in patients with chronic hepatitis B. Hepatology 2002;35(6):1522-7.

23. Xu B, Hu DC, Rosenberg DM, et al. Chronic hepatitis B: a long-term retrospective cohort study of disease progression in Shanghai, China. J Gastroenterol Hepatol 2003;18(12):1345-52.

24. Zipprich A, Garcia-Tsao G, Rogowski S, Fleig WE, Seufferlein T, Dollinger MM. Prognostic indicators of survival in patients with compensated and decompensated cirrhosis. Liver Int 2012;32(9):1407-14.

25. Hui AY, Chan HL, Leung NW, Hung LC, Chan FK, Sung JJ. Survival and prognostic indicators in patients with hepatitis B virus-related cirrhosis after onset of hepatic decompensation. J Clin Gastroenterol 2002;34(5):569-72.

26. Lin X, Robinson NJ, Thursz M, et al. Chronic hepatitis B virus infection in the Asia-Pacific region and Africa: review of disease progression. Journal of gastroenterology and hepatology 2005;20(6):833-43.

27. Hassanipour S, Vali M, Gaffari-Fam S, et al. The survival rate of hepatocellular carcinoma in Asian countries: a systematic review and meta-analysis. EXCLI journal 2020;19:108-30.

28. Unicef. Hepatitis B vaccine price data. 2021 [Access date 9 Oct 2022. Available from: <https://www.unicef.org/supply/documents/hepatitis-b-hepb-vaccine-price-data>.]

29. Murakami H, Van Cuong N, Huynh L, Hipgrave DB. Implementation of and costs associated with providing a birth-dose of hepatitis B vaccine in Viet Nam. Vaccine 2008;26(11):1411-9.

30. Toi PL, Wu O, Thavorncharoensap M, et al. Economic evaluation of population-based type 2 diabetes mellitus screening at different healthcare settings in Vietnam. PLoS One 2021;16(12):e0261231.

31. Due OT, Thakkinstian A, Thavorncharoensap M, et al. Cost-Utility Analysis of Direct-Acting Antivirals for Treatment of Chronic Hepatitis C Genotype 1 and 6 in Vietnam. Value Health 2020;23(9):1180-90.

32. Jia YX, Cui FQ, Li L, et al. Comparison between the EQ-5D-5L and the EQ-5D-3L in patients with hepatitis B. Qual Life Res 2014;23(8):2355-63.
